# Placebo Effects: Neurological Mechanisms Inducing Physiological, Organic, and Belief Responses—A Prospective Analysis

**DOI:** 10.3390/healthcare12222314

**Published:** 2024-11-20

**Authors:** Sarfaraz K. Niazi

**Affiliations:** College of Pharmacy, University of Illinois, Chicago, IL 60612, USA; sniazi3@uic.edu; Tel.: +1-312-297-0000

**Keywords:** placebo trials, randomized control trials, real-world evidence, addictions, organic changes, belief systems, therapeutic applications

## Abstract

The placebo effect can induce physiological or clinical neurological and organic responses despite the recipient receiving no active ingredients; these responses are based instead on the recipient’s perceptions. Placebo effects come from the rostral anterior cingulate cortex, pontine nucleus, and cerebellum of the brain; this information provides a better understanding of placebo effects and can also help us understand the mechanism of the modulation of neurotransmitters from the use of psychedelic substances, activity of selective serotonin reuptake inhibitors, the process of transcranial magnetic stimulation, and deep brain stimulation, as well as aid in developing novel therapies, challenging the validity of controlled clinical trials (RCTs) that the regulatory agencies now appreciate. Education about how placebo effects bring in social, political, and religious beliefs and whether these can be modulated may help reduce global confrontations.

## 1. Introduction

The concept of the placebo has a rich history dating back to ancient times [1]. The term “placebo” comes from the Latin word meaning “I shall please”, and it was used in medieval times to describe sham treatments given to comfort patients. By the 18th century, physicians started recognizing these inert treatments’ psychological effects. Dr. John Haygarth is often credited with conducting one of the first documented placebo-controlled experiments in 1799 in which he demonstrated that placebo treatments could elicit real, measurable effects in patients with rheumatism [2]. A recent study published in *Science Translational Medicine* [3] tested how people reacted to migraine pain medication to show that the placebo was 50% as effective as the analgesic drug once the participants were told that the tested drug could reduce pain. It is now proven that placebos are not inert; they can initiate neural stimulation that create emotional, physiological, and even organic responses, including clinical efficacy.

In the 20th century, placebos in clinical trials became more structured and formalized. The randomized controlled trial [4] design, introduced in the 1940s, incorporated placebos as a control mechanism to evaluate the efficacy of new treatments [5]; the double-blind placebo-controlled trial became the gold standard for clinical research, with claims about the objectivity and reliability of the results being highlighted [6]. However, we have since found that subjective outcomes, such as pain, fatigue, and overall well-being, and objective outcomes, such as clinical response, are highly influenced by the placebo effect. These findings challenge the most widely used random control trial (RCT) protocols that risk rejecting new drugs when compared with placebos despite requiring a substantially larger study size.

In this paper, I present a prospective analysis, as it could be argued, about how placebo effects, despite their little understood etiology, can be used to discover new drugs, rationalize clinical trials, and resolve conflicts by understanding the differences in belief systems.

## 2. Understanding Placebo Effects and Responses

The term “placebo effect” refers to the therapeutic or beneficial outcome a patient experiences when receiving a placebo, which has no active therapeutic ingredient. The effect is attributed to psychological and physiological responses, such as belief in treatment efficacy, expectation of improvement, and conditioned responses. It is more general and encompasses the broad range of benefits that might occur simply from receiving a treatment, regardless of its actual content.

The term “placebo response” is a broader term that includes all the outcomes associated with receiving a placebo in a clinical trial, not only the therapeutic effects but also any adverse effects or subjective experiences. The placebo response covers positive (improvement) and negative (side effects or no change) reactions to the placebo and includes measurable changes in the patients’ symptoms or conditions.

The concepts of placebo and nocebo effects reveal the profound impact that expectations can have on health outcomes, encompassing positive and negative responses. While the placebo effect demonstrates how positive expectations can lead to improvements in symptoms—even when the treatment is inactive—the nocebo effect shows the other side of this phenomenon. In the case of nocebo effects, negative expectations about a treatment or condition can result in adverse outcomes, such as increased pain, nausea, or other unpleasant symptoms. This effect highlights how the mind’s anticipatory mechanisms can influence physical experiences that may worsen a person’s condition, even without a medical cause.

Understanding the nocebo effect expands the scope of mind–body interactions, emphasizing that hope or belief can foster healing, and fear or anxiety can worsen or even create symptoms. For instance, patients who read about potential side effects before taking a new medication may be more likely to experience these side effects, not because of the drug itself but due to their heightened anxiety and anticipation of harm. This phenomenon complicates the administration of medical treatments and presents an ethical challenge for healthcare providers. It raises questions about how much information should be shared with patients to avoid unintentionally triggering nocebo responses and how to prepare patients in a way that mitigates these effects without compromising transparency.

The interplay between placebo and nocebo effects underscores the significance of psychological factors in health and treatment efficacy. By studying both effects, researchers can better understand the full spectrum of mind-body interactions and explore therapeutic strategies that harness positive expectations while minimizing negative ones. For example, educating patients about the potential for positive outcomes while reducing emphasis on minor side effects could enhance treatment experiences and outcomes. In clinical practice, recognizing and addressing nocebo effects can lead to more compassionate, effective care by focusing on the biochemical effects of treatments and managing patients’ perceptions and expectations in ways that promote healing.

**Figure 1:**
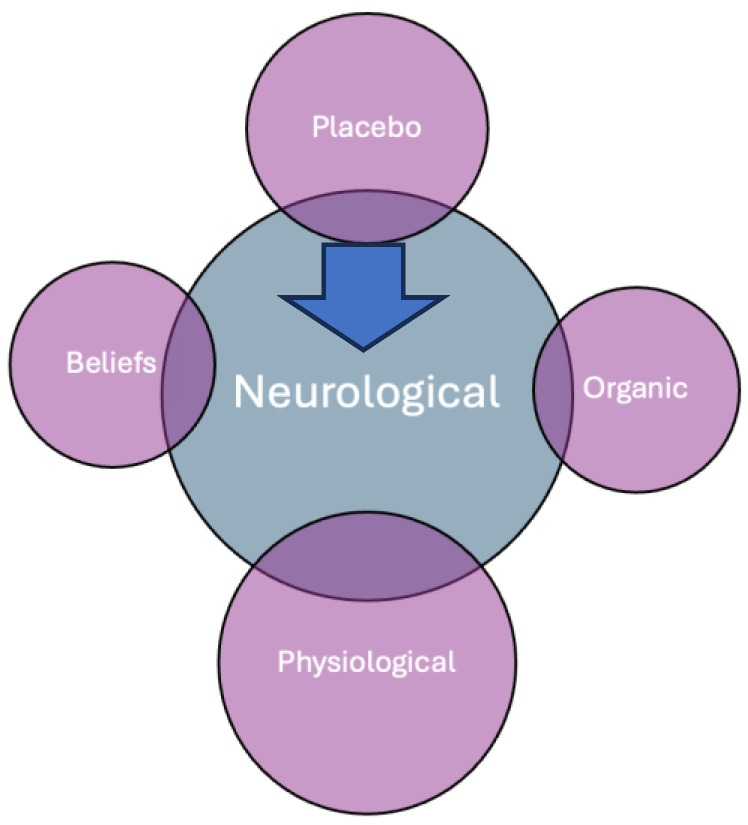
Placebo effects are delivered through neurological systems.

Regardless of the nature of a placebo effect or response, it starts with activating the neurological system, which results with physiological responses like analgesia, organic reactions such as immune suppression, or the formation of beliefs triggered by feelings of security and comfort (Figure 1).

While knowledge of the placebo effect has existed for centuries, the mechanisms that trigger it, maintain its resilience, and create a life-long mindset are still under study. A recent article in *Nature* identified a specific neural circuit involving the rostral anterior cingulate cortex (rACC), pontine nucleus, and cerebellum that reduced pain perception through enhanced neuronal excitability and synaptic plasticity [7]. The rACC plays a pivotal role in mediating this effect. Specifically, neurons projecting from the rACC to the pontine nucleus (Pn), a region involved in cortico-cerebellar communication, were identified as central to the analgesic response. Activating these neurons through optogenetics induced analgesia, even without placebo conditioning, while inhibiting the pathway, which disrupted pain relief.

The active brain regions involved in placebo-induced analgesia were identified using advanced experimental techniques. The researchers first utilized a placebo analgesia conditioning (PAC) assay to create a behavioral model in mice that mimics human placebo analgesia. To monitor neural activity during the anticipation of pain relief, they employed in vivo calcium imaging, which allowed real-time observation of active neurons. Specifically, they focused on neurons in the rACC, a region previously implicated in placebo analgesia from human brain imaging studies. By targeting rACC neurons that project to the Pn, the team used viral tracing methods to label and track neural pathways. Optogenetics, a technique that enables precise control of neuronal activity using light, was employed to activate or inhibit these neural circuits during the conditioning and post-test phases. Additionally, single-cell RNA sequencing of Pn neurons revealed an abundance of opioid receptors, suggesting their role in modulating pain. Combining behavioral assays, calcium imaging, optogenetics, and molecular analysis, this comprehensive approach allowed the researchers to pinpoint the rACC→Pn→cerbellum pathway as a critical mediator of placebo analgesia [6,8].

This definitive proof of placebo intervention extends our understanding of the role of opioids and gamma-aminobutyric acid (GABA) agonists in reducing pain signals. It further supports the mechanisms of non-invasive transcranial magnetic stimulation (TMS) and optogenetic stimulation [9], physical interventions, and non-physical interventions such as mindfulness-based stress reduction (MBSR) and cognitive behavioral therapy (CBT), reframing negative pain-related thoughts by reducing the activity of pain-related neural circuits, including those in the rACC [10,11].

Currently, placebo-based treatments are increasingly recognized for their potential to enhance patient outcomes in various clinical settings, particularly in pain management, mental health, and functional disorders. For example, open-label placebo treatments, in which patients are aware that they are receiving a placebo, have shown promise for conditions like irritable bowel syndrome (IBS) and chronic low back pain. Studies have demonstrated that even when patients know they are taking a placebo, they can still experience significant symptom relief, likely due to the psychological power of expectations and the therapeutic context of care [12]. Another area of application is mental health, particularly in the management of depression and anxiety. Meta-analyses of antidepressant trials often reveal substantial placebo responses, suggesting that patient expectations and the therapeutic alliance between the patient and provider play critical roles in treatment efficacy [13]. Placebos are also used to enhance the effects of real treatments through conditioning; for instance, a patient who has previously experienced relief from a medication may continue to respond to a placebo resembling the medication in appearance [13]. These examples illustrate how placebo-based approaches can be ethically and effectively integrated into modern healthcare, emphasizing the importance of patient perception, expectations, and the clinical environment in achieving therapeutic benefits.

### Quantum Effects

The idea that quantum biology could play a role in placebo effects is speculative and largely theoretical [14]. Quantum processes such as superposition, entanglement, and tunneling have been suggested to influence biological systems in ways that classical physics cannot fully explain. Although there is no strong empirical evidence, some researchers propose that quantum mechanisms might influence the brain’s processing of expectations and perceptions related to placebo responses that can have significant implications in biology [15].

Theories by Stuart Hameroff and Roger Penrose propose that quantum processes within microtubules, structural components of neurons, could contribute to consciousness, as microtubules can support quantum coherence, influencing neural function at a fundamental level [16] where particles exist in multiple states simultaneously, potentially playing a role in complex neural computations and information processing [17].

**Figure 2:**
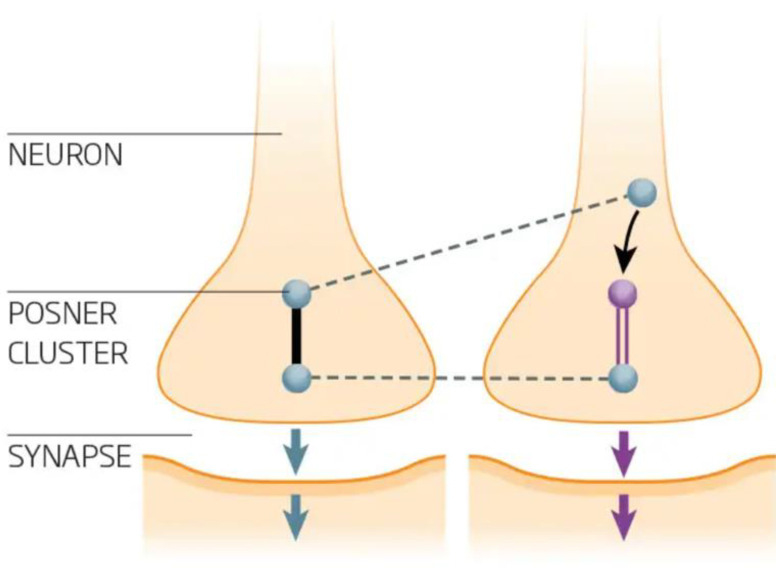
The structure and function of a nerve cell. Entangled Posner clusters initiate chemical signaling which spreads to other neurons by binding to receptors on the neural membrane of dendritic spines, opening ion channels and altering the next cell’s membrane potential; thus, the signal traverses as shown by the dotted lines (Created by BioRender).

The brain contains many cell types, from the prominent neurons to the lesser-known microglia. The latter are integral to the brain’s immune system and play a crucial role as the brain’s cleanup crew. A recent study highlighted that microglia establish connections with neurons through tunneling nanotubes, facilitating the clearance of toxic proteins from the neurons to promote neuronal health [18] (Figure 2).

Quantum theories of consciousness suggest that processes like the observer effect might impact placebo effects. This could theoretically link quantum biology to mind–body interactions, where patient expectations influence physiological outcomes. Other researchers have performed a review of quantum consciousness theories [19].

The idea that quantum coherence or entanglement could influence brain function—and thus placebo responses—comes from speculative models proposing quantum effects at synaptic and neurotransmitter levels. Quantum tunneling is one process hypothesized to play a role in neurotransmitter signaling. Other studies provide more on this topic [20]. Some experimental studies have hinted at quantum effects in biological systems, such as photosynthesis in plants and avian navigation [21]. For example, chlorophyll is almost instantly synthesized from sunlight and carbon sources as the particles pass through thermodynamic barriers [15].

However, most of these ideas remain theoretical and have not been rigorously tested through empirical research. While quantum biology holds promise for expanding our understanding of complex biological systems, including the placebo effect, current scientific evidence does not yet support a definitive role for quantum mechanics in explaining placebo phenomena

## 3. Physiological Responses

Neurobiologically, the most widely studied form of placebo response is placebo analgesia, where patients experience a reduction in pain due to the activation of endogenous opioid systems. This process involves the release of endorphins which bind to opioid receptors in the brain, thereby reducing pain perception. Studies have shown that brain areas such as the anterior cingulate cortex, periaqueductal grey, and amygdala are less active in patients experiencing placebo analgesia, suggesting that these brain regions play key roles in modulating pain and emotional response [22]. In some cases, placebo responses have been shown to persist even when opioid antagonists like naloxone are administered, indicating that non-opioid systems, such as the endocannabinoid system, also contribute to placebo analgesia.

Given the diversity of placebo responses, it is impossible to standardize the impact of a placebo. For example, not all participants receiving a placebo painkiller show a response, and there is no way to standardize this response for any type or class of drug since it is driven by a combination of past experiences, social influences, and cognitive processes [9].

A comparison between a drug in pill form and a device or surgery would be subject to a similar effect if, for example, the overall response to a device or surgery includes a more significant placebo component than the pill to which it is compared. Under such circumstances, the difference between two treatments might result from differences in their placebo effects rather than in the therapies themselves. Suppose the placebo can influence the outcome or interpretation of a therapeutic trial in this way; in such a case, it is essential to determine what factors may modify the “placebo dose” and ask if different sham controls have other effects [23].

Another observation is that the strength of a drug placebo’s response is related to its route of administration. In 1955, Louis Lasagna, an early investigator of the placebo effect, noted that “an injection is thought to be more effective than something taken by mouth” [24]. It was said that “for universal patient acceptance, nothing can approach the psychotherapeutic impressiveness of puncture of the integument by a hollow needle. The placebo substance introduced via the needle is usually second in importance to the needle stick [itself]” [25].

The notion of an “enhanced” [26] or “mega-placebo” [27] effect is probably applicable to interventions such as surgery or devices if, as claimed, the placebo effect is influenced by elaborate rituals [28], appeals to mysterious powers [29], or high technology [30]. Indeed, acceptance of an enhanced placebo effect underlies the opinion that practicing physicians may “choose treatment whose appearance or route of administration is known to be associated with strong placebo effects” [31]. But do we know whether heightened placebo effects exist? As Lasagna states, “the art of the placebo” is “based not on any systematic investigation of the facts but on impressions” [32].

Neuroimaging studies have demonstrated that brain regions such as the anterior cingulate cortex (ACC), the prefrontal cortex, and the brainstem are activated during placebo effects, which modulate pain perception by engaging descending inhibitory pathways.

**Figure 3:**
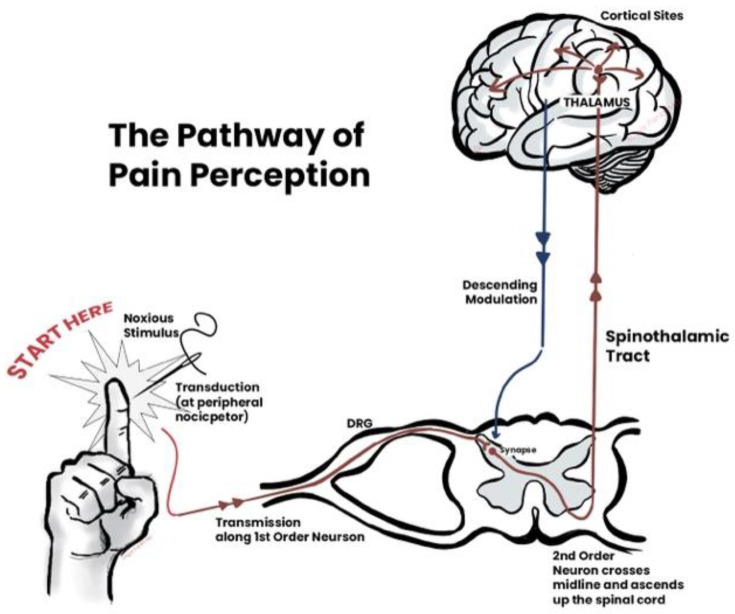
The pathway of pain reception in the brain, known as the pain or nociceptive path, begins with specialized nerve cells called nociceptors located throughout the body in the skin, muscles, joints, and internal organs. When these nociceptors detect harmful stimuli, such as extreme temperatures, pressure, or chemical irritants, they generate electrical signals that travel through sensory nerve fibers. These signals are transmitted to the spinal cord via peripheral nerves and enter the dorsal horn of the spinal cord, where they undergo initial processing. From the spinal cord, the signals travel upward through the spinothalamic tract to the brainstem and ultimately reach the thalamus, which acts as a relay station. The thalamus distributes the pain signals to various regions of the brain, including the somatosensory cortex, which interprets the intensity and location of the pain; the limbic system, which processes the emotional response to pain; and the prefrontal cortex, which is involved in the cognitive assessment of the pain experience. Additionally, the brain sends descending signals to the spinal cord to modulate pain perception, often releasing neuromodulators such as endogenous opioids to reduce pain intensity. This complex network of ascending and descending pathways allows the brain to perceive and respond to pain in a nuanced and integrated manner, involving sensory but also emotional and cognitive dimensions. Placebo analgesia, or pain relief from a placebo, is a complex psychoneurological event triggered by a patient’s belief that they are receiving a medical intervention. Verbal suggestions, contextual cues, or social learning can cause this belief. It can involve the release of neuromodulators such as opioids, cholecystokinin, cannabinoids, and dopamine (Created by BioRender).

Figure 3 shows how the pain placebo response works.

The cannabinoid system, through CB1 receptors, has been shown to mediate pain relief, and blocking these receptors can diminish the analgesic effects of placebos. Dopaminergic pathways, particularly those involved in reward anticipation, also play a role in placebo responses, especially in conditions like Parkinson’s disease. In Parkinson’s patients, placebo treatments have been associated with increased dopamine release in the striatum, which is linked to improvements in motor function. This highlights the role of neurotransmitter systems beyond opioids in mediating placebo responses [33].

Empathy neurons, known as mirror neurons, are specialized brain cells that activate when an individual observes another performing an action or experiencing an emotion, such as pain [34]. These neurons are located primarily within the premotor and parietal cortices and are associated with emotional understanding and empathetic responses. Studies using functional MRI (fMRI) have demonstrated that specific brain regions, such as the anterior insula and the anterior cingulate cortex, are activated when individuals observe others experiencing pain. This shared neural circuitry implies that our brains simulate the observed pain, leading to an empathetic response. By activating similar neural pathways as those involved when experiencing pain firsthand, the brain allows the observer to feel a version of the pain they see in others, facilitating emotional understanding and social bonding [35].

These neurons were first discovered in the premotor cortex of macaque monkeys, where they were observed to fire both when the monkey performed an action and when it observed the same action being performed by another [36]. This mirroring mechanism is not confined to motor actions. Still, it extends to emotions and sensations, suggesting that these neurons play a crucial role in empathy by enabling an individual to understand and share the emotional states of others [37].

## 4. Organic Responses

The placebo effect can produce organic changes in the body, often through physiological mechanisms closely linked to the mind–body interaction. When a person expects a positive outcome from treatment, even if it is inactive (placebo), this expectation can trigger measurable biological responses. One key mechanism is the activation of the brain’s endogenous opioid system. Studies have shown that placebo-induced analgesia, for example, involves the release of endorphins and other natural painkillers in the brain, which can reduce pain perception like that of actual analgesic drugs.

Several recent discoveries have shown that the placebo effect is not limited to subjective experiences like pain relief but extends to measurable neurobiological changes, influencing the immune and endocrine systems. Integrating the impact across different physiological systems could enhance overall well-being and complement conventional medical treatments for various conditions, such as neurological and immune disorders.

Advances in neuroimaging have also revealed the involvement of several neurotransmitter systems, such as the opioid, cannabinoid, and monoaminergic systems, which mediate placebo responses. Additionally, conditioned immune responses have been observed, where regions like the insular cortex and amygdala contribute to immune suppression via noradrenaline. Although the exact molecular mechanisms behind these responses are not fully elucidated, there is compelling evidence that placebo treatments can lead to natural, organic changes in the body that mirror the effects of actual pharmacological interventions [22].

Psychologically, the placebo effect capitalizes on the power of the mind, leveraging mechanisms such as expectation, learning, memory, and motivation. Classical conditioning plays a crucial role, where a neutral stimulus, when paired with an active treatment, can evoke similar biological responses without the active agent. This principle extends to many clinical settings where patients who receive active treatment can also experience enhanced therapeutic benefits from a placebo. The patient–doctor relationship also significantly impacts the placebo response, as verbal cues and reassurance from physicians can amplify the patient’s expectations and thereby boost the efficacy of the placebo. For instance, studies have shown that explicit verbal information can improve the analgesic effects of placebo treatments by heightening patient expectations.

The role of expectation is particularly notable in placebo responses, as patients who expect positive outcomes—due to past experiences, environmental cues, or emotional states—are more likely to experience real improvements in their symptoms. This phenomenon is not merely psychological but is reflected in brain activity, as neuroimaging studies have shown increased activation in brain regions like the prefrontal cortex, anterior insula, and nucleus accumbens during placebo treatments [33]. These regions are integral to emotion, cognition, and reward processing, and their involvement further underscores the tangible nature of placebo effects.

Incorporating the concepts of expectation and conditioning can deepen our understanding of why certain individuals are more susceptible to placebo responses than others. Expectation, which can be explored through expectancy theory, plays a central role in placebo responses by influencing how individuals perceive potential outcomes based on their beliefs and hopes. When people expect a treatment to work, their bodies and minds may respond in ways that mimic the treatment’s intended effect, even if it is inactive. This response is not solely psychological, it can also involve physiological changes, showing the powerful link between mind and body. Conditioning, particularly classical conditioning, also contributes to placebo responses. For example, if a person has previously taken medication that alleviates pain, they may associate the act of taking a pill with pain relief. Over time, this association becomes so strong that simply taking an inert pill can trigger pain relief due to the conditioned response.

Moreover, prior experiences with treatment and broader psychological factors—such as anxiety, depression, or optimism—further shape an individual’s susceptibility to placebo effects. Individuals with higher levels of anxiety or depression may react differently to placebos compared to those with more optimistic or resilient outlooks, suggesting that personality and mental health contribute significantly to placebo responsiveness. Anxiety, for instance, might heighten sensitivity to symptoms, potentially making a person more likely to notice and respond to subtle placebo effects. On the other hand, optimistic individuals may have stronger positive expectations, amplifying the placebo response. Altogether, expectation and conditioning, influenced by personal history and psychological state, create a complex interplay that determines the strength and nature of each person’s placebo response. Understanding this interplay can provide insights into tailoring treatment approaches that harness the power of expectation and conditioning in a therapeutic setting, potentially enhancing overall outcomes.

Furthermore, the placebo effect can influence the autonomic nervous system, altering heart rate, blood pressure, and immune function. For instance, placebo treatments have been shown to reduce inflammation by triggering the release of anti-inflammatory molecules such as cortisol. These effects are mediated through conditioned responses and learned associations, where the body responds to the expectation of relief or healing as if an active treatment were administered. Thus, placebo effects can lead to genuine, organic changes in the body, driven largely by neurobiological processes involving the brain’s reward, pain, and stress regulatory systems.

Since perceptions and beliefs triggered within the brain are chemically dependent, there is great interest in research connecting quantum biology and placebo effects, leading to the possibility that quantum phenomena could influence the neural processes underlying the placebo response.

### 4.1. Homeopathy

Homeopathy was invented in the late 18th century by Samuel Hahnemann, a German physician dissatisfied with the conventional medical practices of his time, which often involved bloodletting and purging. He created the concept of “like cures like”, or *similia similibus curentur*, which posited that substances causing symptoms in healthy individuals could treat similar symptoms in the sick when administered in highly diluted forms [38]. Despite containing no measurable amount of any drug, he believed these dilutions could retain a “memory” of the substance, an idea that remains a cornerstone of homeopathic practice. Homeopathy gained popularity in Europe and the United States during the 19th century as a gentler alternative to the harsh medical treatments of the era. By the mid-19th century, numerous homeopathic institutions had been established, including hospitals and medical schools, most notably the New York Homeopathic Medical College, founded in 1860 [38].

Homeopathy is widely considered a placebo treatment due to its lack of efficacy beyond placebo effects. Numerous systematic reviews and meta-analyses have found that homeopathic treatments do not perform better than placebos [39,40]. Major health organizations, including the U.K. House of Commons Science and Technology Committee [41] and the Australian National Health and Medical Research Council [41], have reviewed the evidence and concluded that homeopathy lacks scientific plausibility and efficacy [40,42]. It continues to be practiced by many people worldwide, with varying degrees of regulation and acceptance across different countries [43]. In India, homeopathic practitioners often hold a Bachelor of Homeopathic Medicine and Surgery (BHMS) and must be licensed and registered [43]. European regulations vary, with some countries requiring formal training and certification. Indian studies argue for the clinical efficacy of homeopathic treatments [43].

### 4.2. Meditation

Mindfulness meditation has also been found to reduce the perception of pain and improve the quality of life of patients with chronic pain [44]. Both yoga and Tai Chi, which incorporate mindfulness and physical movement, benefit chronic pain conditions [44]. Meditation practices, including mindfulness and T.M., have been associated with improvements in cardiovascular health [42] and working memory capacity. Meditation increases the cortical thickness of the brain regions involved in sensory, cognitive, and emotional processing [45].

### 4.3. Simulator Therapies

Addictions to substances like nicotine, alcohol, and opioids are deeply tied to the brain’s reward system, primarily through the release of dopamine, which reinforces pleasurable behaviors. Current pharmacological treatments aim to mimic or block these effects, reducing dependency. For instance, nicotine replacement therapies (NRT) like patches and gums help reduce cravings by delivering lower doses of nicotine without the harmful chemicals in tobacco, while opioid agonists such as methadone and buprenorphine target opioid receptors to reduce cravings and withdrawal symptoms [46].

Methadone, a full agonist, and buprenorphine, a partial agonist, bind to opioid receptors in the brain but provide less intense effects than drugs like heroin or oxycodone. Buprenorphine, in particular, has a safer side effect profile, making it especially useful for patients with severe addiction, though methadone has been found more effective in treatment retention [47]. To reduce opioid cravings without risk of overdose, buprenorphine is often combined with naloxone, an opioid antagonist, which blocks euphoric effects and lowers the risk of abuse [47].

Meanwhile, naltrexone, another antagonist, is used to prevent relapse in both opioid and alcohol addiction by blocking the receptors that would otherwise create pleasurable effects [48].

## 5. Beliefs

Another significant aspect of the placebo is its role in influencing how humans develop beliefs, mostly embedded by societal influence. The neural underpinnings of various belief systems involve a core set of regions in the prefrontal cortex, anterior temporal lobe, and limbic areas [49]. From a cognitive perspective, humans have an innate tendency to detect patterns, which can lead to belief in supernatural entities such as gods and spirits, that may have helped early humans survive by remaining cautious of potential threats [50]. Cognitive scientists suggest these mechanisms make religious beliefs intuitive and compelling, contributing to their persistence [49,50].

The formation of beliefs related to religion, ideologies, politics, and racism is deeply intertwined with neural mechanisms involving expectation, reward, and emotional regulation [51]. These placebo effects are also linked to resiliency mechanisms partially determined by genetic factors [52]. Neuroimaging studies have demonstrated that psychotherapy and conscious emotional regulation can significantly influence brain activity [49], highlighting the importance of mentalistic variables in understanding human behavior and brain functioning.

### 5.1. Religion

Belief systems are deeply rooted in neural mechanisms that involve expectation, reward processing, emotional regulation, and social cognition. Understanding the brain regions and circuits involved in these processes, we can understand why certain beliefs become resilient and how social and emotional factors can influence them. This knowledge has profound implications for addressing issues such as religious extremism, political polarization, and racial prejudice, as it highlights the importance of targeting the underlying neural processes involved in belief formation and maintenance [53].

The placebo effect starts with feeling satisfied, forming belief systems that remove or attenuate the stress of uncertainty. One such example is the creation of the concept of God. The idea of someone out there watching you is comforting, as shown by more than 80% of the world following an ideological god. The same holds for political and cultural ideologies that form bonds among groups promising to support each other. This ideological pursuit can extend to discrimination as one means of differentiation, thus creating homogeneous groups that serve as the basis of racist, nationalist, and condescending attitudes that are well-established in civilized societies.

Religious beliefs are triggered by the temporal–parietal junction (TPJ), which processes beliefs about the intentions of others and aids in understanding their perspectives, a key component in religious thought and practice and theory of mind [54]. The medial prefrontal cortex (PFC) is associated with self-referential thinking and is active when reflecting on personal beliefs and values. This area integrates personal experiences with abstract religious concepts. Regions such as the amygdala and hippocampus within the limbic system are involved in the emotional aspects of religious experiences. These areas process the emotional intensity and memories associated with religious rituals and experiences [51].

Interestingly, the circuits have been identified as having a role in analgesia have also been recognized to have a role in belief systems [55]. Religious beliefs can enhance analgesia through emotional detachment, engaging the right ventrolateral prefrontal cortex [56]. This is best exemplified by self-flagellation, a disciplinary and devotional practice of flogging oneself with whips or other instruments that inflict pain, prescribed in all Abrahamic religions practiced since the Egyptian and Greco-Roman cults [57]. Remarkably, the feeling of pain goes away when the belief system supersedes the nerve transmissions. There is no better example of how the mind can control the sensation of pain.

Unlike clinical trial placebos, which rely on the patient’s belief in a treatment, religious beliefs are deeply rooted in human cognition, social structures, and cultural traditions. Thus, while both involve significant psychological components, the driving forces behind religious belief are more diverse and complex. The neural circuits that govern expectation and reward processing are critical players in pain modulation and belief formation, suggesting a shared neurological basis for these complex cognitive phenomena [58].

Promoting religion also has a vital role in the placebo effect, involving spreading the word of the benefits of the afterlife, being surrounded by people of similar belief, and, in most illiterate societies, a feeling of responsibility for the task. More people have been killed due to religious wars than any other cause; this shows how the significance, resilience, and power of this placebo effect.

### 5.2. Ideologies

Ideological beliefs, including political, economic, and social ideologies, are often reinforced by group identity and shared values. The prefrontal cortex and limbic system are involved in processing group cohesion and identity, which are crucial for maintaining ideological beliefs. Cognitive biases and social influences further shape these beliefs, with neural mechanisms responding to social validation and dissonance resolution. For instance, brain imaging studies have shown that political beliefs can activate areas associated with emotional regulation and social cognition, indicating the complex interplay of neural processes in forming and defending ideologies [59]. The neural mechanisms of group cohesion and identity processing in the prefrontal cortex and limbic system play a significant role here. Individual values, social identity, and cognitive biases shape political beliefs. The brain’s response to political information involves emotional regulation and cognitive dissonance, influencing how political ideologies are adopted and defended [60].

Humans tend to favor information that supports their pre-existing beliefs, known as confirmation bias. This cognitive bias strengthens political beliefs by filtering and interpreting information in a way that aligns with one’s political ideology, much like how belief in the efficacy of a placebo can lead to perceived health improvements [61]. Furthermore, political beliefs often fulfill emotional and psychological needs by providing a framework for social identity and group belonging.

The brain regions that trigger political beliefs and religious beliefs are the same. The dorsolateral prefrontal cortex (DLPFC) is crucial for executive functions such as reasoning, decision-making, and cognitive control [59]. It plays a pivotal role in maintaining and processing political beliefs, especially when individuals are confronted with information that contradicts their beliefs [62].

Emotional regulation also plays a significant role in belief formation. Beliefs often provide emotional comfort or stability, engaging brain regions such as the amygdala and anterior cingulate cortex. These regions are also involved in the placebo response, suggesting a shared pathway for emotional regulation through belief. Cognitive dissonance, the discomfort of holding conflicting beliefs or encountering contradictory evidence, activates the anterior cingulate cortex. This neural response drives individuals to resolve dissonance by reinforcing their beliefs or modifying them to reduce conflict [63].

Ideological beliefs are rooted in a deep confidence in following the only correct path; this explains why such stark differences can exist. The placebo effect triggered by feelings of superiority over others, such as those found in Nazi groups, is a clear example of how an ideology can be turned into a tool against others.

### 5.3. Discrimination

Discrimination, including racism, shares similarities with the placebo effect in terms of how beliefs influence perceptions and behaviors, providing a false sense of comfort and identity. The psychological comfort derived from discriminatory beliefs can be likened to the placebo effect, in which expectations shape perceptions. Individuals holding racist beliefs often experience a sense of superiority and security, reinforcing their worldview and identity, much like how belief in a placebo treatment can lead to perceived improvements in health [64].

Racist beliefs can stem from in-group/out-group biases processed in the amygdala and prefrontal cortex. Social conditioning and emotional responses to perceived threats or stereotypes reinforce these beliefs, highlighting the roles of social influence and emotional regulation in their formation. Understanding these mechanisms can provide insight into why certain beliefs are resilient and how social and emotional factors influence them. This knowledge has profound implications for addressing issues such as religious extremism, political polarization, and racial prejudice by targeting the underlying neural processes involved in belief formation and maintenance [58].

Discrimination has similar roots to other ideological placebos. The most significant forms of discrimination are based on race, skin color, religious belief, gender, and other elements are embedded in the 30 articles of the United Nations Declaration of Human Rights [65], which is rarely followed by all who have signed it. In the U.S., Title VI of the Civil Rights Act of 1964 is a perfect reminder that discrimination is illegal. Still, this need for reminders is necessary because of placebo effects that inevitably lead us to discriminate against others [66]. However, despite such acknowledgments and countless attempts to remove discrimination, it remains one of the strongest placebo effects, giving believers a sense of superiority triggered by addictive dopamine responses.

## 6. Placebo Prospective Analysis

Our better understanding of placebo effects, albeit still conjectural in many cases, requires projecting how the present drugs can be better put to use, how the new drugs are tested more logically, and what clinical interventions can be designed based on the placebo effect.

### 6.1. Clinical Applications

Looking forward, research is focusing on more targeted approaches, such as dopamine modulators and Corticotropin-Releasing Factor (CRF) antagonists, which may help manage stress-induced cravings, a key factor in addiction relapse [46].

Furthermore, psychedelics such as psilocybin show promise in reprogramming the brain’s reward circuits, potentially helping to reduce dependence and offering longer-lasting recovery by altering the way individuals perceive their addictions. By addressing both the biological and psychological aspects of addiction, future therapies could combine these pharmacological innovations with advanced neuroscience to create more personalized and effective treatments [48].

Although dopamine itself cannot be directly administered, dopamine supplements are available in forms that can boost dopamine production or enhance its function within the brain. L-tyrosine, an amino acid, is one of the most widely used supplements. It is a precursor to L-DOPA, which is further converted into dopamine, supporting cognitive performance and mood regulation, especially in stressful conditions. This supplement has been studied for its ability to mitigate cognitive fatigue and maintain dopamine levels during intense physical or mental exertion [47].

L-DOPA, often in the form of levodopa, is used primarily in the treatment of Parkinson’s disease. It crosses the blood–brain barrier and is converted directly into dopamine, effectively replenishing the diminished dopamine levels typical in Parkinson’s patients. This supplement is highly effective in managing the disease’s motor symptoms, though long-term use may lead to diminished efficacy. Mucuna pruriens, a natural source of L-DOPA, has been shown to have similar effects as synthetic levodopa and is gaining popularity as a natural supplement for dopamine support in managing conditions like Parkinson’s [47].

Another supplement, SAM-e (S-Adenosyl Methionine), contributes to dopamine synthesis and has been investigated for its potential to alleviate depression by supporting neurotransmitter production, including dopamine. Supplementation with SAM-e has been shown to improve mood and cognitive function, particularly in individuals with depression.

Alongside these supplements, Vitamin B6 is also essential for dopamine synthesis, acting as a cofactor in converting L-DOPA to dopamine. Low levels of Vitamin B6 can reduce dopamine production, making its supplementation crucial for maintaining balanced neurotransmitter levels [48].

Additionally, Omega-3 fatty acids, commonly found in fish oil, play a significant role in supporting the structure and function of neurons, including those that release dopamine. Omega-3 supplementation has been linked to improved cognitive function and mood, likely due to its ability to enhance dopamine receptor function and signaling. Curcumin, the active ingredient in turmeric, has been studied for its neuroprotective properties. Animal studies have demonstrated its potential to increase dopamine levels, and it has gained attention as a supplement for mental health and mood regulation.

In terms of pharmaceutical interventions, dopamine agonists such as pramipexole and ropinirole directly stimulate dopamine receptors, offering benefits in treating Parkinson’s disease and restless leg syndrome by compensating for the loss of natural dopamine signaling. Similarly, monoamine oxidase inhibitors (MAOIs), which prevent the breakdown of dopamine, serotonin, and norepinephrine, are used to increase the availability of these neurotransmitters, particularly in the treatment of depression and neurodegenerative diseases [47,48].

The future of dopamine-related therapies may involve genetic techniques like CRISPR, which could potentially enhance dopamine synthesis directly within the brain, or novel compounds that better target dopamine receptors with fewer side effects [67]. Moreover, substances such as psilocybin, currently under investigation for their effects on dopamine receptors, could represent a breakthrough in neuropsychiatric treatments, offering the potential for long-term dopamine modulation [68].

By leveraging natural supplements and pharmaceutical agents, dopamine levels can be managed effectively for mood, cognitive function, and neurodegenerative disease management, with ongoing research promising even more targeted and personalized interventions.

Dopamine cannot be administered directly as a treatment for several reasons, primarily due to its inability to cross the blood–brain barrier (BBB). The blood–brain barrier is a selective membrane that protects the brain from potentially harmful substances in the bloodstream, but it also prevents many helpful molecules, like dopamine, from entering. Dopamine is a relatively large molecule, and the tight junctions in the BBB do not allow it to pass through.

To bypass this limitation, treatments for conditions like Parkinson’s disease use L-DOPA (Levodopa), a precursor to dopamine. L-DOPA can cross the blood–brain barrier, where it is then converted into dopamine by an enzyme called aromatic L-amino acid decarboxylase. This indirect method allows dopamine to be replenished in the brain without directly administering it.

Using transferrin as a carrier to deliver dopamine across the blood–brain barrier (BBB) is a promising concept due to the receptor-mediated transcytosis mechanism that transferrin utilizes. The transferrin receptor is abundantly expressed on endothelial cells in the BBB, making it a potential vehicle for transporting therapeutic agents like dopamine into the brain. Once bound to transferrin, the dopamine could theoretically hitch a ride across the BBB, utilizing the transferrin receptor to enter the brain, where it could then be released to exert its therapeutic effects. This strategy has been employed in various drug delivery systems targeting the brain, including small molecules, peptides, and nanoparticles [69]. Despite these challenges, transferrin-mediated delivery remains a promising approach, particularly in treating neurodegenerative diseases where increased dopamine in the brain could be beneficial. Continued research into refining this strategy, especially regarding complex stability, release mechanisms, and receptor saturation, could open new avenues for delivering dopamine and other therapeutic agents across the BBB [70].

No therapies are specifically designed to change complex belief systems like religion, political ideologies, or discrimination. However, some therapies, albeit not ethically intended for this, can influence cognitive and emotional processes that might indirectly impact these systems. Psychedelic drugs, such as LSD, psilocybin (found in magic mushrooms), and MDMA, have been shown to temporarily alter perception and reduce rigid thinking, possibly leading to an increased openness to new ideas. Some users of these substances report long-term shifts in their worldviews following therapeutic sessions, though their ability to permanently alter core beliefs remains debatable [71].

Brain stimulation therapies, including transcranial magnetic stimulation (TMS) and deep brain stimulation (DBS), can target brain regions associated with decision-making and moral reasoning. These have been studied for their effects on reducing ideological rigidity by altering neural activity, though their impact on fundamental belief systems is modest [72].

Similarly, neurofeedback and cognitive-behavioral interventions focus on training individuals to control brain activity and reframe extreme thought patterns, which might reduce bias or rigidity but do not guarantee a change in deeply held beliefs [73].

Pharmacological agents like oxytocin, which promotes social bonding, have shown some capacity to reduce group-based biases, including discrimination. However, these effects are context-dependent and do not directly alter complex belief systems [74].

Memory modulation therapies, like using propranolol to reduce emotional responses to traumatic memories, may influence how people process past events but are unlikely to lead to significant shifts in ideologies or religion [75].

While these methods can influence cognitive and emotional flexibility, their capacity to directly and permanently alter complex belief systems like religion or political ideology is limited. Furthermore, the ethical implications of using such therapies to influence beliefs are significant, raising concerns about autonomy and the potential for misuse [76].

The ethical implications of placebo use in medical and psychological treatments are complex and widely debated, particularly when considering the differences between deceptive placebos and open-label placebos. Deceptive placebos involve administering an inactive treatment without the patient’s knowledge, allowing them to believe they are receiving effective therapy. This approach raises ethical concerns primarily around patient autonomy and informed consent. Deception can undermine trust between patients and practitioners, compromising the transparency that is foundational to ethical medical practice. Critics argue that withholding information from patients, even to achieve a therapeutic effect, violates their right to make informed decisions about their treatment. Additionally, there is the risk that patients who later discover they were given a placebo may feel misled, potentially leading to negative psychological effects and decreased trust in medical professionals.

In contrast, open-label placebos offer a more ethically transparent approach. Patients are informed that they are receiving a placebo, yet research has shown that these placebos can still elicit positive therapeutic responses. This outcome may be due to expectation and conditioning, as patients who understand the potential benefits of placebos may still experience symptom relief. The ethical advantage of open-label placebos lies in their honesty, as patients are fully aware of what they are receiving. Supporters of open-label placebos argue that this approach respects patient autonomy while allowing individuals to benefit from the placebo effect in a way that maintains trust in the patient-provider relationship. However, some critics question whether the therapeutic effects of open-label placebos are strong enough to justify their use, especially in cases where effective, evidence-based treatments are available.

Thus, the ethical debate surrounding placebos centers on the balance between potential therapeutic benefits and the need for transparency and informed consent. While deceptive placebos may produce stronger effects in certain contexts, they carry a significant ethical burden. Open-label placebos, while ethically sounder, may not be as effective in every case. This ongoing ethical debate highlights the importance of further research to understand when and how placebos can be used in a way that respects patient autonomy while maximizing therapeutic outcomes.

### 6.2. Clinica Efficacy Testing

RCTs are the gold standard for establishing the safety and efficacy of new drugs; however, recent research on the impact of placebo in these trials suggests otherwise. In trials where the placebo has a definitive perception or organic effect, the target drug will be considered less effective, a significant drawback of placebo–control studies. These studies raise an issue of ethics, as critically ill patients receiving a placebo will be disadvantaged [77].

As with RCTs [78], experience with trials conducted in clinical practice settings has demonstrated the potential value of this approach for drug development in certain circumstances. Such trials with simplified data collection have allowed rapid enrollment and evidence generation [79]. More recently, the widespread use of electronic health records (EHRs) and other electronic data-gathering tools has made integrating clinical research and care more feasible. For example, in 2020, the RECOVERY trial was conducted using clinical practice infrastructure and local HCPs in hospitals throughout the United Kingdom. The trial results supported FDA approval of tocilizumab for treating hospitalized adult patients with COVID-19 who received systemic corticosteroids and required supplemental oxygen, noninvasive or invasive mechanical ventilation, or extracorporeal membrane oxygenation [80].

#### 6.2.1. Statistics

Another reason for removing placebo controls is the inevitable increase in study size. Such studies are biased due to their smaller size, making them more subjective [81]. The FDA requires a study power of 80% and α of 0.05 for comparative clinical trials such as those conducted to prove biosimilarity [82,83]; however, in almost all such studies, the study power was significantly low, resulting in all such studies being positive. While several other agencies like the MHRA and EMA consider such trials uncertain and unnecessary, the FDA must take a similar action.

**Figure 4:**
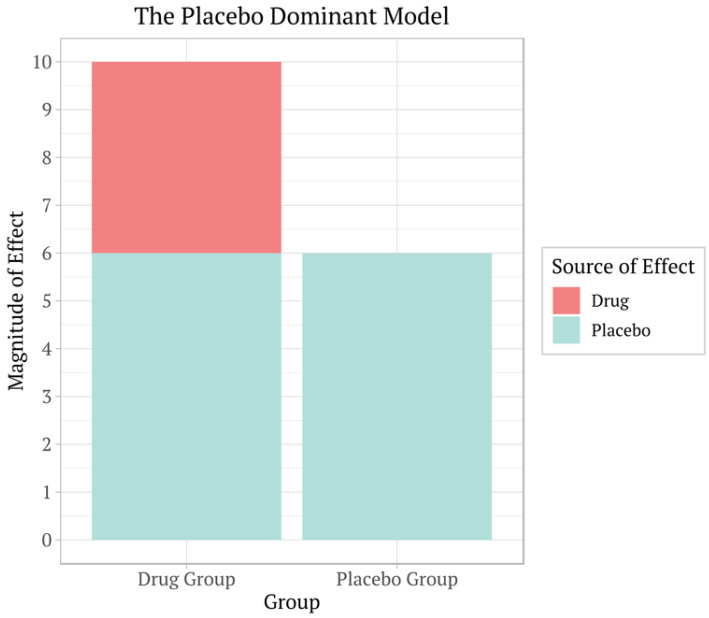
Clinical trials often compare the effects of a drug to a placebo to determine the drug’s effectiveness and check for side effects. In these trials, participants are randomly assigned to either receive the drug or an inactive placebo and are usually unaware of which they are receiving. This is called a double-blind test, and it helps keep the research free from bias. By comparing the results from both groups, researchers can measure how the drug works and see if it is more effective than the placebo effect alone. Placebo effects can easily make a drug’s effects less prominent should it be approved for its gross effect and not the net effect. There can also be a nonlinear response if the placebo effect is subtracted, which cannot be estimated, downgrading the effective response (Hypothetical drawing by the author).

A systematic review and meta-analysis of 12,564 men found a significant association between placebo use and improved erectile function, with the effect size being larger among men with post-traumatic stress disorder [84]. Continuous outcomes, such as pain intensity measured on a scale, show more pronounced placebo effects than binary outcomes, such as the presence or absence of a symptom. This difference indicates that placebos might influence how patients perceive and report their symptoms rather than causing actual physiological changes [85]. Studies using three-armed trials [81] provide more precise insights into the placebo effect by isolating it through direct comparisons between placebo and no-treatment groups. These studies’ results suggest that placebos can noticeably impact patient-reported outcomes. Nevertheless, the effects are often modest and can diminish with larger sample sizes and more rigorous methodologies [81]. However, it remains uncertain what the correct sample size is to overcome the complexity of the placebo effect (Figure 4).

The nonlinearity of the placebo response was demonstrated when an analysis was made of the RCTs that examined the effectiveness of H2 blockers; it was found that drug effectiveness was demonstrated only in those studies whose placebo responses were relatively small. In other words, the results of these trials depended on the magnitude of the response to the placebo and the response to the active drug.

#### 6.2.2. Ethics

The ethical implications of using placebos in clinical trials have been a topic of considerable debate. Regulatory agencies, including the World Medical Association and the U.S. Food and Drug Administration (FDA), have recommended avoiding placebo use in certain situations, primarily for ethical reasons. The Declaration of Helsinki, first adopted in 1964 and subsequently revised, emphasizes that placebos should not be used when effective treatments are available, as this could deny patients the best possible care [86]. The ethical argument against placebos hinges on beneficence and non-maleficence, ensuring that patients are not subjected to unnecessary harm or deprived of necessary treatment [86]. In addition, the conduct of RCTs brings several dilemmas, as shown in Table 1.

The only utility of a placebo control is to establish that the response to treatment is more than the placebo; a creative approach is to test the placebo first in a small population; if no placebo effect is noted, then the need for the placebo arm can be removed. When a placebo response is recorded, the treatment must have a higher response based on stand-alone studies. This acceptance criterion will be challenged; however, once we realize that, unlike homeopathy, regulatory agencies cannot approve a placebo as a drug if the placebo response is high, a slight addition will merely serve an ethical purpose. If a placebo response is established, the acceptance criteria can be established above the placebo effect. Since a comparative statistic is unnecessary, the study size for efficacy testing will be significantly reduced.

The FDA and other agencies are now suggesting the adoption of RWE, where the safety and efficacy are established in a protocol that matches the post-approval use of the tested drug, removing two elements: randomization and control, as these are never a part of RWE use of a drug. If the tested drugs are subject to a patient-driven placebo effect, then how the drug is administered in an RWE condition can significantly vary the placebo effect. This suggestion is closer to the FDA’s newest guideline issued in September 2024 that brings the RCTs into the domain of RWE [87,88,89,90,91].

## 7. Implications for Future Therapies

The understanding of placebo and nocebo effects has significant implications for the development of future therapies, impacting both drug development and patient management strategies. As we explore the role of patient expectations in therapeutic outcomes, it becomes evident that these psychological effects can be strategically incorporated to enhance drug efficacy and improve patient adherence. For instance, in drug development, researchers can leverage the knowledge of the placebo effect to design studies that better account for the psychological influence on patient outcomes, particularly in trials for pain management, mental health, and chronic conditions [92]. By understanding the mechanisms behind placebo responses, pharmaceutical developers can refine how they present potential patient benefits, fostering positive expectations that may complement the drug’s therapeutic action. Similarly, awareness of the nocebo effect has important implications in patient management; clinicians can minimize adverse responses by carefully framing information about side effects, as patients who anticipate negative effects are more likely to experience them [93]. This “expectation management” strategy could be especially useful in fields like oncology and rheumatology, where strong medications often come with significant side effects, and patients’ expectations about these can directly influence their quality of life and treatment adherence [94].

Moreover, incorporating placebo and nocebo effects into therapeutic strategies could shift the treatment paradigm from a solely biomedical approach to one that integrates psychological and contextual factors in patient care. This could involve training healthcare professionals to communicate potential benefits effectively while addressing concerns in a way that reduces anxiety, thereby limiting the likelihood of nocebo responses. Such patient-centered approaches may improve health outcomes by strengthening trust and patient confidence in treatment plans [95]. Research has also suggested that therapies incorporating patient expectancy could reduce drug dosages without compromising efficacy, as the placebo response amplifies perceived therapeutic effects [96]. Drug developers and clinicians should consider the placebo and nocebo effects not as biases to be eliminated but as therapeutic tools that, when understood and applied appropriately, can lead to more effective, personalized, and holistic patient care. Integrating these effects into clinical practice and drug development can ultimately support a future of medicine that respects the complex interplay between mind and body, with the potential to improve both efficacy and patient satisfaction across a range of treatments.

## 8. Conclusions

A placebo intervention can bring about more than changes in perception; it can lead to organic changes mediated by the brain and genuine physiological changes impacting disease symptoms and progression. This dual mechanism underscores the complexity and power of the placebo effect in clinical practice and research. The placebo effect primarily involves the disease course in the context of infections and cancer. In oncology, placebos mainly address symptoms and side effects rather than altering the disease course [97] because the concept of placebos affecting organic components is still unexplored. The brain’s release of endogenous opioids and other neurotransmitters in response to placebo treatments can modulate pain pathways, providing genuine pain relief that goes beyond mere perception. Additionally, placebo effects can activate brain regions associated with stress and immune responses, potentially influencing the body’s physiological state and improving symptoms [98].

For a long time, the organic impact of belief has been widely disputed. Now that we can understand how quantum tunneling can lead to organic changes in the brain, the subject of quantum biology is about to move beyond its infancy. Based on our current knowledge, several changes in how drugs are discovered and tested, how good health is maintained, and how the brain can transform humanity are proposed.

Drug development technology has undergone vast changes, from anecdotal medicine to the AI-driven discovery of hit molecules. We have entered a new era, but now we need to include quantum biology in the picture. Can we manipulate quantum tunneling to produce organic effects? Can we train microglia to identify brain cancer cells? Can we find an antidote to addictions that, in turn, are created by quantum tunneling in the brain? We present a new perspective on manipulating the body’s systems to treat diseases. The prospects are wide-ranging, but it is a practical and rational choice given the proof of its functionality.

This concept has been explored in the context of brain function and neural processes. Yet, many neuroscientists and physicists are skeptical, arguing that the brain’s warm, wet, and noisy environment is not conducive to maintaining quantum coherence, asserting that classical physics sufficiently explains neural processes [99]. More experimental studies are needed to test the hypothesis of quantum tunneling in neural processes, utilizing advanced imaging and molecular techniques. Improved theoretical models integrating quantum mechanics with neuroscience could better aid the understanding of how quantum phenomena might influence brain functions. Collaboration between physicists, neuroscientists, and biologists is crucial to advance understanding. Thus, while quantum tunneling in brain function holds the potential to revolutionize our understanding of neural processes and consciousness, significant scientific challenges remain.

Clinical trial designs must be modified significantly to ensure we are not unnecessarily exposing human subjects. The regulatory agencies have developed propositions for substituting the CRTs, which will substantially facilitate the development of new drugs. Do drugs show an effect when they are infinitely diluted, as claimed by homeopathy? Which chemicals are secreted in the brain when someone sacrifices their life for a belief or cause? Can we find drugs that will trigger the same neurons as when we pray or engage in self-flagellation? The doors are now wide open for a new era of quantum biology, and like the quantum properties of particles, so are our predictions; they could be everywhere. We need not see them; we just need to believe in them.

Placebo effects leading to addictions, both conceptual and organic, can be treated by drugs or mental training once their role is established in a patient’s personality. Many beliefs are strong enough to lead to radicalism; can we bring moderation by prescribing chemical drugs that can desensitize individuals who have become dependent on the dopamine rushes? Alcohol and drug addictions are another possibility for treating dependence by supplementing with chemical drugs that can moderate the need for these surges.

The field of placebo effects shall remain uncertain because of the uncertainty of the mechanisms involved in the responses related to placebo. Still, we know these are real and can be exploited in developing and testing new drugs with more rational approaches that prevent ethical risks and provide a more optimal path to discoveries.

## Figures and Tables

**Table 1:** Comparison of events in RCTs vis-à-vis placebos.

Efficacy of Drug	Efficacy of Placebo	Statistics	Resolution
+++, ++, +	+++, ++, +	Non-significant	Reject. Only homeopathy has addressed this; no agency will approve such drugs.
0	0	Non-significant	Rejected.
+++	++	Significant	Approved based on difference as a response.
+	++	Significant	Rejected based on additional effects of the drug.
+++, ++, +	Placebo arm removed	Significant	A much smaller study size reduced unnecessary exposure to humans.

## Data Availability

Not applicable.

## References

[1] Beecher H.K. (1955). The powerful placebo. J. Am. Med. Assoc..

[2] Haygarth J. (2023). Of the Imagination as a Cause and Cure of Disorders of the Body, Exemplified by Fictitious Tractors.

[3] Kam-Hansen S., Jakubowski M., Kelley J.M., Kirsch I., Hoaglin D.C., Kaptchuk T.J., Burstein R. (2014). Altered placebo and drug labeling changes the outcome of episodic migraine attacks. Sci. Transl. Med..

[4] Ratz-Łyko A., Arct J. (2019). Resveratrol as an active ingredient for cosmetic and dermatological applications: A review. J. Cosmet. Laser Ther..

[5] Gupta U., Verma M. (2013). Placebo in clinical trials. Perspect. Clin. Res..

[6] Miller F.G., Colloca L., Kaptchuk T.J. (2009). The placebo effect: Illness and interpersonal healing. Perspect. Biol. Med..

[7] Lazar S.W., Kerr C.E., Wasserman R.H., Gray J.R., Greve D.N., Treadway M.T., McGarvey M., Quinn B.T., Dusek J.A., Benson H. (2005). Meditation experience is associated with increased cortical thickness. Neuroreport.

[8] Chen C., Niehaus J.K., Dinc F., Huang K.L., Barnette A.L., Tassou A., Shuster S.A., Wang L., Lemire A., Menon V. (2024). Neural circuit basis of placebo pain relief. Nature.

[9] Eippert F., Bingel U., Schoell E.D., Yacubian J., Klinger R., Lorenz J., Büchel C. (2009). Activation of the opioidergic descending pain control system underlies placebo analgesia. Neuron.

[10] Lozano A.M., Lipsman N., Bergman H., Brown P., Chabardes S., Chang J.W., Matthews K., McIntyre C.C., Schlaepfer T.E., Schulder M. (2019). Deep brain stimulation: Current challenges and future directions. Nat. Rev. Neurol..

[11] Petrovic P., Kalso E., Petersson K.M., Ingvar M. (2002). Placebo and opioid analgesia—Imaging a shared neuronal network. Science.

[12] Kaptchuk T.J., Friedlander E., Kelley J.M., Sanchez M.N., Kokkotou E., Singer J.P., Kowalczykowski M., Miller F.G., Kirsch I., Lembo A.J. (2010). Placebos without deception: A randomized controlled trial in irritable bowel syndrome. PLoS ONE.

[13] Fournier J.C., DeRubeis R.J., Hollon S.D., Dimidjian S., Amsterdam J.D., Shelton R.C., Fawcett J. (2010). Antidepressant drug effects and depression severity: A patient-level meta-analysis. JAMA.

[14] Weingarten C.P., Doraiswamy P.M., Fisher M.P. (2016). A New Spin on Neural Processing: Quantum Cognition. Front. Hum. Neurosci..

[15] Lambert N., Chen Y.-N., Cheng Y.-C., Li C.-M., Chen G.-Y., Nori F. (2013). Quantum biology. Nat. Phys..

[16] Scheiblich H., Eikens F., Wischhof L., Opitz S., Jüngling K., Cserép C., Schmidt S.V., Lambertz J., Bellande T., Pósfai B. (2024). Microglia rescue neurons from aggregate-induced neuronal dysfunction and death through tunneling nanotubes. Neuron.

[17] Frömer R., Nassar M.R., Ehinger B.V., Shenhav A. (2024). Common neural choice signals can emerge artefactually amid multiple distinct value signals. Nat. Hum. Behav..

[18] Wager T.D., Atlas L.Y. (2015). The neuroscience of placebo effects: Connecting context, learning and health. Nat. Rev. Neurosci..

[19] Hameroff S., Penrose R. (2014). Consciousness in the universe: A review of the ‘Orch OR’ theory. Phys. Life Rev..

[20] Fisher M.P.A. (2015). Quantum cognition: The possibility of processing with nuclear spins in the brain. Ann. Phys..

[21] Tegmark M. (2000). Importance of quantum decoherence in brain processes. Phys. Rev. E.

[22] Oken B.S. (2008). Placebo effects: Clinical aspects and neurobiology. Brain.

[23] Kaptchuk T.J., Goldman P., Stone D.A., Stason W.B. (2000). Do medical devices have enhanced placebo effects?. J. Clin. Epidemiol..

[24] Liberman R. (1962). An analysis of the placebo phenomenon. J. Chronic Dis..

[25] Chaput de Saintonge D.M., Herxheimer A. (1994). Harnessing placebo effects in health care. Lancet.

[26] Kaptchuk T.J. (1998). Powerful placebo: The dark side of the randomised controlled trial. Lancet.

[27] Roberts A.H., Kewman D.G., Mercier L., Hovell M. (1993). The power of nonspecific effects in healing: Implications for psychosocial and biological treatments. Clin. Psychol. Rev..

[28] Vickers A.J. (1998). Bibliometric analysis of randomized trials in complementary medicine. Complement. Ther. Med..

[29] Taub H.A., Mitchell J.N., Stuber F.E., Eisenberg L., Beard M.C., McCormack R.K. (1979). Analgesia for operative dentistry: A comparison of acupuncture and placebo. Oral. Surg. Oral. Med. Oral. Pathol..

[30] Hashish I., Ho Kee H., Harvey W., Feinmann C., Harris M. (1988). Reduction of postoperative pain and swelling by ultrasound treatment: A placebo effect. Pain.

[31] Koes B.W., Scholten R.J.P.M., Mens J.M.A., Bouter L.M. (1995). Efficacy of epidural steroid injections for low-back pain and sciatica: A systematic review of randomized clinical trials. Pain.

[32] Johnson A.G. (1994). Surgery as a placebo. Lancet.

[33] Ortega Á., Salazar J., Galban N., Rojas M., Ariza D., Chávez-Castillo M., Nava M., Riaño-Garzón M.E., Díaz-Camargo E.A., Medina-Ortiz O. (2022). Psycho-Neuro-Endocrine-Immunological Basis of the Placebo Effect: Potential Applications beyond Pain Therapy. Int. J. Mol. Sci..

[34] Hartmann H., Rütgen M., Riva F., Lamm C. (2021). Another’s pain in my brain: No evidence that placebo analgesia affects the sensory-discriminative component in empathy for pain. Neuroimage.

[35] Singer T., Seymour B., O’Doherty J., Kaube H., Dolan R.J., Frith C.D. (2004). Empathy for pain involves the affective but not sensory components of pain. Science.

[36] Rizzolatti G., Craighero L. (2004). The mirror-neuron system. Annu. Rev. Neurosci..

[37] Keysers C., Gazzola V. (2009). Expanding the mirror: Vicarious activity for actions, emotions, and sensations. Curr. Opin. Neurobiol..

[38] Greenberg S.J. (1986). On the history of New York Medical College. N. Y. Med. Q..

[39] Shang A., Huwiler-Müntener K., Nartey L., Jüni P., Dörig S., Sterne J.A., Pewsner D., Egger M. (2005). Are the clinical effects of homoeopathy placebo effects? Comparative study of placebo-controlled trials of homoeopathy and allopathy. Lancet.

[40] Cucherat M., Haugh M.C., Gooch M., Boissel J.P. (2000). Evidence of clinical efficacy of homeopathy. A meta-analysis of clinical trials. HMRAG. Homeopathic Medicines Research Advisory Group. Eur. J. Clin. Pharmacol..

[41] House of Commons Science and Technology Committee—Fourth Report. Evidence Check 2: Homeopathy. https://publications.parliament.uk/pa/cm200910/cmselect/cmsctech/45/4502.htm.

[42] Cramer H., Lauche R., Haller H., Dobos G. (2013). A systematic review and meta-analysis of yoga for low back pain. Clin. J. Pain..

[43] India National Commission for Homoeopathy. https://www.nch.org.in.

[44] Varanasi R., Srivastava A., Kumar Rt S., Bala R. (2023). Practice, prescription habits, experience and perception of Indian homeopathic practitioners in treatment of diabetes mellitus: An online observational study. J. Ayurveda Integr. Med..

[45] Schneider R.H., Grim C.E., Rainforth M.V., Kotchen T., Nidich S.I., Gaylord-King C., Salerno J.W., Kotchen J.M., Alexander C.N. (2012). Stress reduction in the secondary prevention of cardiovascular disease: Randomized, controlled trial of transcendental meditation and health education in Blacks. Circ. Cardiovasc. Qual. Outcomes.

[46] Piske M., Thomson T., Krebs E., Hongdilokkul N., Bruneau J., Greenland S., Gustafson P., Karim M.E., McCandless L.C., Maclure M. (2020). Comparative effectiveness of buprenorphine-naloxone versus methadone for treatment of opioid use disorder: A population-based observational study protocol in British Columbia, Canada. BMJ Open.

[47] Maria Carmen B.-G., Sandra M.-R., Marta R.-A., Pilar Almela R. (2019). Present and Future Pharmacological Treatments for Opioid Addiction. Opioids.

[48] Noble F., Marie N. (2019). Management of Opioid Addiction With Opioid Substitution Treatments: Beyond Methadone and Buprenorphine. Front. Psychiatry.

[49] Tannenholz L., Jimenez J.C., Kheirbek M.A. (2014). Local and regional heterogeneity underlying hippocampal modulation of cognition and mood. Front. Behav. Neurosci..

[50] van Prooijen J.W., van Vugt M. (2018). Conspiracy Theories: Evolved Functions and Psychological Mechanisms. Perspect. Psychol. Sci..

[51] van Elk M., Aleman A. (2017). Brain mechanisms in religion and spirituality: An integrative predictive processing framework. Neurosci. Biobehav. Rev..

[52] Peciña M., Zubieta J.-K. (2014). Molecular Mechanisms of Placebo Responses in Humans. Mol. Psychiatry.

[53] Scheffer M., Borsboom D., Nieuwenhuis S., Westley F. (2022). Belief traps: Tackling the inertia of harmful beliefs. Proc. Natl. Acad. Sci. USA.

[54] Kapogiannis D., Barbey A.K., Su M., Zamboni G., Krueger F., Grafman J. (2009). Cognitive and neural foundations of religious belief. Proc. Natl. Acad. Sci. USA.

[55] Kaplan J.T., Gimbel S.I., Harris S. (2016). Neural correlates of maintaining one’s political beliefs in the face of counterevidence. Sci. Rep..

[56] Wiech K., Farias M., Kahane G., Shackel N., Tiede W., Tracey I. (2008). An fMRI study measuring analgesia enhanced by religion as a belief system. PAIN.

[57] Geoffrey A. Flagellation. Encyclopedia Britannica. https://www.britannica.com/topic/flagellation.

[58] Timmann D., Drepper J., Frings M., Maschke M., Richter S., Gerwig M., Kolb F.P. (2010). The human cerebellum contributes to motor, emotional and cognitive associative learning. A review. Cortex.

[59] Asp E., Ramchandran K., Tranel D. (2012). Authoritarianism, religious fundamentalism, and the human prefrontal cortex. Neuropsychology.

[60] Jost J.T., Baldassarri D.S., Druckman J.N. (2022). Cognitive–motivational mechanisms of political polarization in social-communicative contexts. Nat. Rev. Psychol..

[61] Nickerson R.S. (1998). Confirmation Bias: A Ubiquitous Phenomenon in Many Guises. Rev. Gen. Psychol..

[62] Amodio D.M., Jost J.T., Master S.L., Yee C.M. (2007). Neurocognitive correlates of liberalism and conservatism. Nat. Neurosci..

[63] Izuma K., Matsumoto M., Murayama K., Samejima K., Sadato N., Matsumoto K. (2010). Neural correlates of cognitive dissonance and choice-induced preference change. Proc. Natl. Acad. Sci. USA.

[64] Bellavite P., Conforti A., Piasere V., Ortolani R. (2005). Immunology and homeopathy. 1. Historical background. Evid. Based Complement. Altern. Med..

[65] United Nations Universal Declaration of Human Rights. https://www.un.org/en/about-us/universal-declaration-of-human-rights.

[66] United States Congress House (1986). A Bill to Establish a Biotechnology Science Coordinating Committee to Address Scientific Problems Caused by Genetically-Engineered Organisms and a Biotechnology Science Research Program to Support Research and Regulation of the Biotechnology Sciences; to Regulate the Release of Genetically-Engineered Organisms into the Environment and the Use of Such Organisms in Manufacturing and Agricultural Activities, and for Other Purposes.

[67] Tai M.D.S., Gamiz-Arco G., Martinez A. (2024). Dopamine synthesis and transport: Current and novel therapeutics for parkinsonisms. Biochem. Soc. Trans..

[68] Siegel J.S., Subramanian S., Perry D., Kay B.P., Gordon E.M., Laumann T.O., Reneau T.R., Metcalf N.V., Chacko R.V., Gratton C. (2024). Psilocybin desynchronizes the human brain. Nature.

[69] Faresjö R., Lindberg H., Ståhl S., Löfblom J., Syvänen S., Sehlin D. (2022). Transferrin Receptor Binding BBB-Shuttle Facilitates Brain Delivery of Anti-Aβ-Affibodies. Pharm. Res..

[70] Kakinen A., Jiang Y., Davis T.P., Teesalu T., Saarma M. (2024). Brain Targeting Nanomedicines: Pitfalls and Promise. Int. J. Nanomed..

[71] The Lancet Regional Health—Europe (2023). Psychedelic-assisted psychotherapy: Hope and dilemma. Lancet Reg. Health Eur..

[72] D’Onofrio V., Manzo N., Guerra A., Landi A., Baro V., Määttä S., Weis L., Porcaro C., Corbetta M., Antonini A. (2023). Combining Transcranial Magnetic Stimulation and Deep Brain Stimulation: Current Knowledge, Relevance and Future Perspectives. Brain Sci..

[73] Tosti B., Corrado S., Mancone S., Di Libero T., Rodio A., Andrade A., Diotaiuti P. (2024). Integrated use of biofeedback and neurofeedback techniques in treating pathological conditions and improving performance: A narrative review. Front. Neurosci..

[74] Triana-Del Rio R., Ranade S., Guardado J., LeDoux J., Klann E., Shrestha P. (2022). The modulation of emotional and social behaviors by oxytocin signaling in limbic network. Front. Mol. Neurosci..

[75] De Gregorio D., Aguilar-Valles A., Preller K.H., Heifets B.D., Hibicke M., Mitchell J., Gobbi G. (2021). Hallucinogens in Mental Health: Preclinical and Clinical Studies on LSD, Psilocybin, MDMA, and Ketamine. J. Neurosci..

[76] Pearce M.J., Koenig H.G., Robins C.J., Nelson B., Shaw S.F., Cohen H.J., King M.B. (2015). Religiously integrated cognitive behavioral therapy: A new method of treatment for major depression in patients with chronic medical illness. Psychotherapy.

[77] (2013). World Medical Association Declaration of Helsinki: Ethical principles for medical research involving human subjects. JAMA.

[78] FDA Real-World Data: Assessing Electronic Health Records and Medical Claims Data to Support Regulatory Decision-Making for Drug and Biological Products. https://www.fda.gov/regulatory-information/search-fda-guidance-documents/real-world-data-assessing-electronic-health-records-and-medical-claims-data-support-regulatory.

[79] Eapen Z.J., Lauer M.S., Temple R.J. (2014). The imperative of overcoming barriers to the conduct of large, simple trials. JAMA.

[80] RECOVERY Collaborative Group (2021). Tocilizumab in patients admitted to hospital with COVID-19 (RECOVERY): A randomised, controlled, open-label, platform trial. Lancet.

[81] Millum J., Grady C. (2013). The ethics of placebo-controlled trials: Methodological justifications. Contemp. Clin. Trials.

[82] Niazi S. (2022). Scientific Rationale for Waiving Clinical Efficacy Testing of Biosimilars. Drug Des. Devel Ther..

[83] Niazi S.K. (2023). Support for Removing Pharmacodynamic and Clinical Efficacy Testing of Biosimilars: A Critical Analysis. Clin. Pharmacol. Drug Dev..

[84] Koog Y.H., We S.R., Min B.I. (2011). Three-armed trials including placebo and no-treatment groups may be subject to publication bias: Systematic review. PLoS ONE.

[85] Stridh A., Pontén M., Arver S., Kirsch I., Abé C., Jensen K.B. (2020). Placebo Responses Among Men With Erectile Dysfunction Enrolled in Phosphodiesterase 5 Inhibitor Trials: A Systematic Review and Meta-analysis. JAMA Netw. Open.

[86] Howick J., Friedemann C., Tsakok M., Watson R., Tsakok T., Thomas J., Perera R., Fleming S., Heneghan C. (2013). Are treatments more effective than placebos? A systematic review and meta-analysis. PLoS ONE.

[87] FDA Conducting Clinical Trials With Decentralized Elements Guidance for Industry, Investigators, and Other Interested Parties. https://www.fda.gov/media/167696/download.

[88] FDA Good Clinical Practice. https://www.fda.gov/science-research/clinical-trials-and-human-subject-protection/regulations-good-clinical-practice-and-clinical-trials.

[89] FDA Clinical Trials and Human Subject Protection. https://www.fda.gov/science-research/science-and-research-special-topics/clinical-trials-and-human-subject-protection.

[90] FDA FDA Issues Draft Guidance on Conducting Multiregional Clinical Trials in Oncology. https://www.fda.gov/news-events/press-announcements/fda-issues-draft-guidance-conducting-multiregional-clinical-trials-oncology.

[91] FDA Conducting Clinical Trials With Decentralized Elements. https://www.fda.gov/regulatory-information/search-fda-guidance-documents/conducting-clinical-trials-decentralized-elements.

[92] Enck P., Benedetti F., Schedlowski M. (2008). New insights into the placebo and nocebo responses. Neuron.

[93] Amanzio M., Palermo S. (2019). Pain Anticipation and Nocebo-Related Responses: A Descriptive Mini-Review of Functional Neuroimaging Studies in Normal Subjects and Precious Hints on Pain Processing in the Context of Neurodegenerative Disorders. Front. Pharmacol..

[94] Barsky A.J., Saintfort R., Rogers M.P., Borus J.F. (2002). Nonspecific medication side effects and the nocebo phenomenon. JAMA.

[95] Kirsch I. (2019). Placebo Effect in the Treatment of Depression and Anxiety. Front. Psychiatry.

[96] Colloca L., Miller F.G. (2011). Role of expectations in health. Curr. Opin. Psychiatry.

[97] Matarèse B.F.E., Rusin A., Seymour C., Mothersill C. (2023). Quantum Biology and the Potential Role of Entanglement and Tunneling in Non-Targeted Effects of Ionizing Radiation: A Review and Proposed Model. Int. J. Mol. Sci..

[98] Chvetzoff G.l., Tannock I.F. (2003). Placebo Effects in Oncology. JNCI J. Natl. Cancer Inst..

[99] Fleming G., Scholes G., Cheng Y.-C. (2011). Quantum effects in biology. Procedia Chem..

